# Environmental *Toxocara* spp. presence in crowded squares and public parks from San Juan Province, Argentina: A call for a “One Health” approach

**DOI:** 10.3389/fmed.2023.1102396

**Published:** 2023-02-17

**Authors:** Héctor Gabriel Avila, Leonardo Sandon, Paola Emilce Anes, Sergio Andrés Meli, Gustavo Adolfo Giboin, Verónica Mirtha Pérez, María Victoria Periago

**Affiliations:** ^1^Laboratorio Provincial de Zoonosis de San Juan, Ministerio de Salud Pública de San Juan, San Juan, Argentina; ^2^Consejo Nacional de Investigaciones Científicas y Técnicas, Buenos Aires, Argentina; ^3^Facultad de Ciencias Veterinarias, Universidad Católica de Cuyo, San Luis, Argentina; ^4^Fundación Mundo Sano, Buenos Aires, Argentina; ^5^Programa Provincial de Control de Vectores de San Juan, Ministerio de Salud Pública de San Juan, San Juan, Argentina; ^6^Sección de Rabia y Zoonosis, Dirección de Epidemiología, Ministerio de Salud Pública de San Juan, San Juan, Argentina

**Keywords:** *Toxocara canis*, soil transmitted helminths, One Health, spatial epidemiology, San Juan, Argentina

## Abstract

**Introduction:**

Canine soil-transmitted helminth (cSTH) parasites need specific environmental conditions to complete their life cycle. *Toxocara canis* and *T. cati* are the most important zoonotic cSTH, since they are the causal agents of human toxocariasis. Canine STHs are dispersed in feces from infected domestic and wildlife canines. In this study, the presence of STH in canine feces was evaluated in 34 crowded public parks and squares from San Juan Province (Argentina).

**Methods:**

Fecal samples were collected during different seasons in 2021–2022 and analyzed by standard coprological methods, including Sheather and Willis flotation and Telemann sedimentation. InfoStat 2020, OpenEpi V. 3.01 and R and RStudio® were used for statistical analysis and QGIS 3.16.10 for mapping.

**Results:**

From a total of 1,121 samples collected, 100 (8.9%) were positive for at least one intestinal parasite (IP) and three cSTH species were detected: *Toxocara* spp., *Toxascaris leonina* and *Trichuris vulpis*. The most prevalent cSTH species was *T. vulpis* (64/1121; 0.057%), while the least prevalent was *Toxocara* spp. (19/1121; 0.017%). The detection of *Toxocara* spp. eggs was significantly different depending on the season. The geo-spatial variation of each cSTH per season is described.

**Discussion:**

This is the first study in San Juan Province to identify environmental contamination of cSTHs in public areas. The specific localization of areas with the presence of cSTH eggs could provide information to guide strategies to reduce the cSTH infection burden in dogs and promote serological screening of the human population for *Toxocara* spp. Given the zoonotic nature of *Toxocara* spp. We hope this information will help to reinforce activities of control programs, focusing on the “One Health” approach.

## 1. Introduction

The One Health approach recognizes that the health of humans, domestic and wild animals, and the wider environment (including ecosystems) are closely linked and interdependent; the term aims to sustainably balance and optimize the overall health of our planet and its inhabitants (1). The approach mobilizes multiple sectors, disciplines and communities at varying levels of society to work together to foster well-being and tackle threats to health and ecosystems, while addressing the collective need for clean water, energy and air, safe and nutritious food, taking action on climate change, and contributing to sustainable development (2). The One Health approach supports global health security by collaboration and communication at the human-animal-environment interface to address shared health threats such as zoonotic diseases, and others. The zoonotic parasitic diseases transmitted by dogs´ feces are considered under this approach, since it interferes with animal and human health, and its propagation generally occurs in the environment (3). In addition, the concept of one health contemplates the consequences produced by climate change (1), a determining factor in the transmission of canine soil-transmitted helminths (cSTH).

The high number of free-roaming dogs found in urban areas can serve as a source of pathogens which may be dangerous to humans; dogs can act as definitive hosts for a high number of parasites (3), some of which are considered zoonotic because they can cause disease in humans. *Toxocara canis*, *Toxocara cati*, and *Ancylostoma caninum* are, respectively, the primary species of zoonotic cSTHs. Other species of non-zoonotic cSTHs could also be present, e.g., *Toxascaris leonina*, and *T. vulpis* (4, 5), although they are not dangerous for humans, they do have an effect on animal health. For this reason, epidemiological studies aid in determining the parasitological status of the population, parasite burden and potential risk areas (6).

Toxocariasis is a parasitic disease transmitted usually from dogs and/or cats that are infected with *T. canis* and *T. cati* (3), to humans. Hosts include cats, dogs, foxes, coyotes, and wolves. These hosts harbor the nematodes in their guts, shedding the eggs in their feces. The embryonated eggs remain infectious for years outside the host. In the wild, carnivorous animals such as cats and dogs consume infected meat or simply soil containing the eggs, and the parasite persists in their gut. Additionally, transplacental transmission has been documented in dogs and cats (7, 8).

In general, individuals infected with these species are asymptomatic, but some develop clinical syndromes which include visceral larva migrans (VLM), ocular larva migrans (OLM), neurotoxocariasis (NT) and covert/common toxocariasis (CT) and can associate with allergic, neurological and/or visual disorders, or cognitive and intellectual deficits in children (9). Recent epidemiological research has estimated that ~1.4 billion people worldwide (10), particularly in subtropical and tropical regions, are infected with, or exposed to *Toxocara* species, indicating that human toxocariasis is a neglected tropical disease (NTD). Diagnosis in humans is based on clinical, epidemiological, and serological data. Indirect IgG ELISA is a widely used serological method for toxocariasis and western blots can be used to confirm positive ELISA findings to reduce false-positive results (11).

Embryonated *Toxocara* spp. eggs in the environment are considered as the most important source of human toxocariasis. These eggs, however, are also a source of infection for definitive and paratenic hosts (12). To become infective, *Toxocara* spp. eggs need specific conditions of temperature and soil (13, 14), which are present in public squares and parks from different tropical and subtropical countries (15). In this study, canine fecal samples from different parks and squares from San Juan Province, Argentina, were analyzed. The samples were collected during the four seasons (autumn, winter, spring, and summer) in each selected area, with the aim to estimate the association between seasons, weather, presence of cSTH eggs and zoonotic risk.

## 2. Methodology

### 2.1. Study area

This study was carried out in the urban area of San Juan Capital (−31.54, −68.52), in the homonymous province; the main squares and parks were included. This area encompasses a surface of 239.12 km^2^. It has about 450,000 inhabitants with a population density of approximately 1880 inhabitants/km^2^. The elevation of the city is ~650 m and it is located in a valley at the eastern border of the Andes Mountain range. According to the Köppen-Geiger climate classification (16, 17), San Juan has an arid climate (BWh/BWk), with low rainfall (<20 mm on any given month), significant diurnal and annual temperature variation (ranging from an average of 32°C in January to 8°C in July), while the average annual temperature is 18°C. The most populated areas were selected for sampling.

### 2.2. Sample collection and coprological analysis

During 2021 and 2022, fresh canine fecal samples from each square and park were collected during each season: autumn (Epidemiological Week – EW – 21 of 2021/May), winter (EW 32/2021/August), spring (EW 45/2021/November) and summer (EW 8/2022/February). The entire samples were collected in pre-labeled plastic bags and subsequently inactivated at −20°C for 2 weeks. Each sample was homogenized, and 10 grams were used and processed using three different concentration methods; two different flotation techniques, Sheather method (saturated sugar solution, 1.25 specific gravity) and Willis method (saturated NaCl solution, 1.20 specific gravity) as well as a sedimentation technique (Telemann method) (18). The techniques chosen for this study are standard concentration techniques that increase the chances of detecting intestinal parasitic structures, including helminth parasites such as *Toxocara* spp. Each sample was microscopically examined at 100× and 400× magnifications. The identification of *Toxocara* spp. eggs was performed using morphological reference (19). Samples were classified as positive if the presence of eggs was confirmed (20).

### 2.3. Statistical analysis

This is a descriptive, cross-sectional, and observational study. The aim was exploratory and descriptive, focused on finding possible associations between the presence of cSTH, specifically *Toxocara* spp. and location and characteristics of the squares and the seasons. The association was examined through *χ*^2^ tests, using InfoStat® V.19 software (21). The parasitic prevalence was calculated and their association with season, department and square/park was analyzed. The Risk Ratio (RR) and Odds Ratio (OR), with 95% Confidence Interval (CI), of statistically significant associations were obtained using OpenEpi V. 3.01 (22).

To explore the distribution characteristics of the most prevalent parasites found, a calculation between observed and expected cases was performed assuming a uniform distribution of positive cases, using R and RStudio®.

### 2.4. Spatial analysis

Given the low number of *Toxocara* spp. positive cases found during the study, a correlation analysis was performed using only *Toxocara* spp. positive parks (*N* = 12). The correlation between its presence and a composite remote sensed index, which can be identified as a proxy for tree shadow, was analyzed. This new index, specifically created for this study, was named the Tree Magnitude Index (TMI) and it is calculated through the multiplication of the Topographic Index Position (TPI) obtained from a Digital Surface Model (DSM) and the Normalized Difference Vegetation Index (NDVI) obtained from satellite imagery. The TMI was treated both as a response variable and as an explanatory one, using the difference between the observed and expected value of positive cases, assuming a homogeneous distribution. High TPI values suggest surface objects that stand out from their surroundings; high NDVI values suggest vigorous vegetation. As a result, when these two factors are multiplied, high values of TMI would indicate high-rise vegetation, such as trees, whereas low values would indicate low and flat lands with little to no vegetation. TMI might therefore be thought of as a tree magnitude index and as a proxy for tree shadows. Only positive NDVI values were taken into consideration to prevent positive outcomes brought on by both negative indices.

The DSM used had a 5 m resolution, generated from a photogrammetric aerial survey by the Argentinian IGN (23). The bandwidth for TPI was 100 m. NDVI was retrieved from Google Earth Engine (24), and it was computed using Sentinel 2 Level-2A imagery (10 m spatial resolution); the values of the image represent the analysis time-span average. The values of TPI (resampled at 10 m) and NDVI were extracted for each pixel, then the product of the two terms was calculated, and finally, the average value of the multiplication of these two indices was computed for each square and park.

### 2.5. Weather data analysis

Weather data from the nearest weather station, San Juan Airport (12 km east of the city center) (25), was retrieved to gauge weather conditions during the 4 weeks of analysis. Seven variables were retrieved: mean temperature (daily average temperature), Diurnal Temperature Variation (DTV), accumulated precipitation, air humidity, cloud cover, solar energy, and wind speed. For every variable, a value for each of the 4 weeks of the analysis was quantified. Values were the weekly average of the rolling mean of the previous 21 days, except for precipitation data, which was the weekly mean accumulation of the previous 21 days. This range was selected due to previous reports stating there were no significant differences detected in the viability of eggs until day 21 (20); maximum infectivity of larvae eggs has been reported up to day 30 of incubation (26, 27).

## 3. Results

### 3.1. Sample collection and coprological analysis

**Figure 1:**
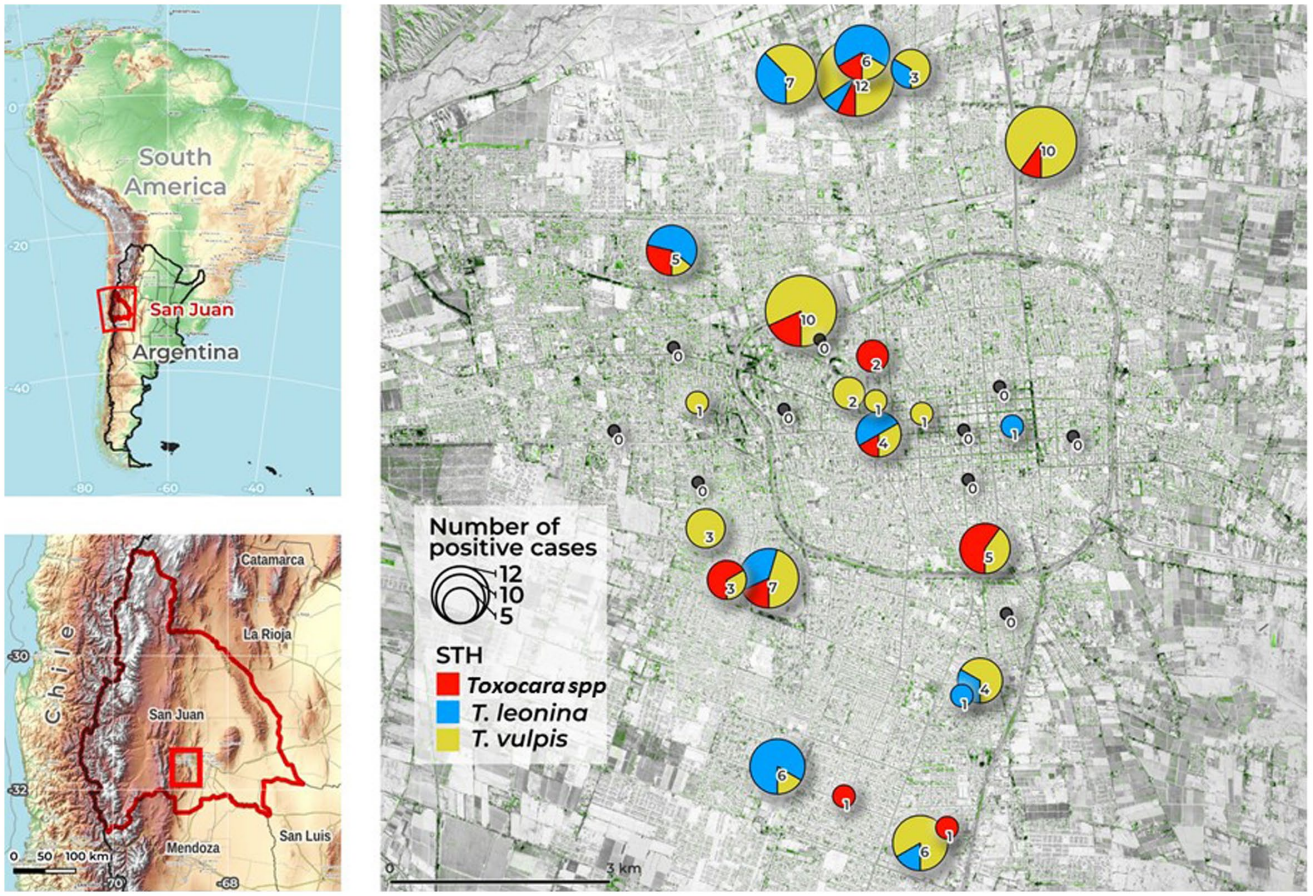
Study area of San Juan City (San Juan, Argentina) in the context of South America. Main Map: Cumulative number of canine soil-transmitted helminths (cSTH) found in each sampling location (2021–2022) by species. Map backgrounds: main map from ©2022 Google; inset map from ©OpenStreetMap, ©OpenTopoMap (CC-BY-SA). Map data: August 09, 2022.

After less than a year of sampling, 1,121 samples were collected, 271 samples in Autumn, 280 samples in Winter, 342 samples in Spring and 228 samples in the Summer. In 8.9% (100/1121) of them, at least one type of cSTH (*Toxocara* spp., *T. vulpis* and *T. leonina*) was found. *Toxocara* spp. eggs were detected in 0.017% (19/1121) of the samples (Table 1). With respect to the other cSTHs found, the most prevalent species was *Trichuris vulpis* (0.0571%; 64/1121), followed by *Toxascaris leonina* (0.0259%; 29/1121). Twelve samples showed almost one type of co-infection (Table 1). Figure 1 shows the overall number of positive samples of the three STH found per sampling location and the general study area that was included in this study. During the study, 67.6% (23/34) of the squares and parks sampled showed environmental contamination with at least one type of cSTH. The presence of *Toxocara* spp. was detected in 12 of the analyzed squares and parks (35.3%).

**Table 1:** Description of the canine fecal samples collected, prevalence of canine soil-transmitted helminths (cSTH) found in total and per season in San Juan City, San Juan, Argentina (2021–2022).

	Autumn (*N* = 271)	Winter (*N* = 280)	Spring (*N* = 342)	Summer (*N* = 228)	Total (*N* = 1,121)	*χ*^2^; d.f. = 3	*p*
Parasite presence (%)	28 (10.33%)*	32 (11.43%)**	25 (7.31%)	15 (6.58%)	100 (8.9%)	5.46	0.1410
*Trichuris vulpis* presence (%)	13 (4.80%)	24 (8.57%)	16 (4.68%)	11 (4.82%)	64 (5.7%)	5.69	0.1279
*Toxascaris leonina* presence (%)	11 (4.06%)	9 (3.21%)	6 (1.75%)	3 (1.32%)	29 (2.6%)	5.17	0.1597
*Toxocara* spp. presence (%)	12 (4.43%)	3 (1.07%)	3 (0.88%)	1 (0.44%)	19 (1.7%)	16.34	<0.001

* There were eight samples which presented co-infection: three between *T. vulpis* and *T. leonina* and 2 between *T. vulpis* and *Toxocara* spp.

** There were four samples which presented co-infection: three between *Toxocara* spp. and *T. leonina*, 3 between *T. vulpis* and *T. leonina* and 1 between *T. vulpis* and *Toxocara* spp.

### 3.2. Statistical and spatial analysis

**Figure 2:**
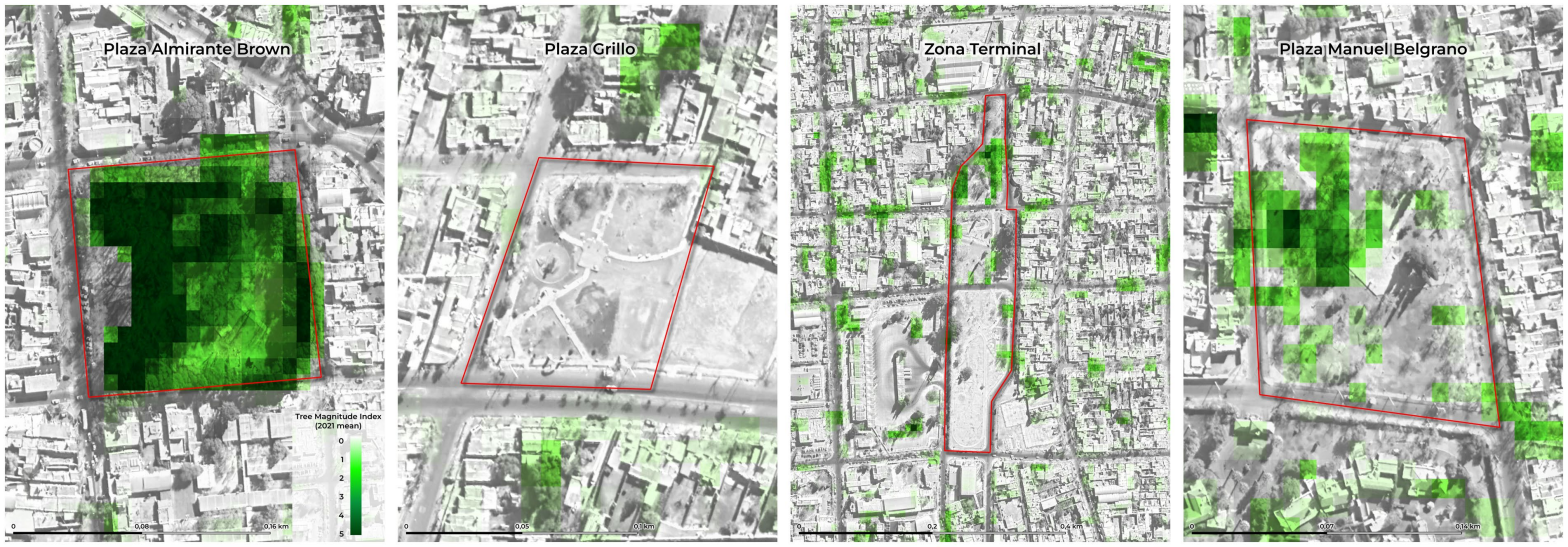
Mean Tree Magnitude Index (TMI) for four of the 34 sampled squares from San Juan City (San Juan, Argentina).

The distribution of cSTHs per season was not uniform, being *T. vulpis* the most prevalent in the samples collected in the autumn, winter, and summer, with *T. leonina* being the most prevalent in the spring. The highest prevalence of *Toxocara* spp. was observed in autumn (4.43%; *p* < 0.01); while there was no statistically significant difference in the prevalence of the other cSTH per season (Table 1). Moreover, the risk and odds ratio analysis showed that there is 5 times greater risk of finding *Toxocara* spp. in dog fecal samples in the autumn compared to the other three seasons (RR = 5.38, 95% CI 2.14–13.5; OR = 5.58, 95% CI: 2.17–14.32). Using simple linear regression, the TMI significantly predicted *Toxocara* spp. prevalence (*R*^2^ = 0.67, *F*(1, 10) = 23.2, *p* < 0.01), with the following fitted regression model: Δ Observed-Expected Value = 0.54 + 0.81*(TMI). Figure 2 shows the mean TMI during 2021 in four of the sampled areas. TMI of the entire study area of San Juan City (San Juan, Argentina) during 2021 is shown in Supplementary Figure 1.

**Figure 3:**
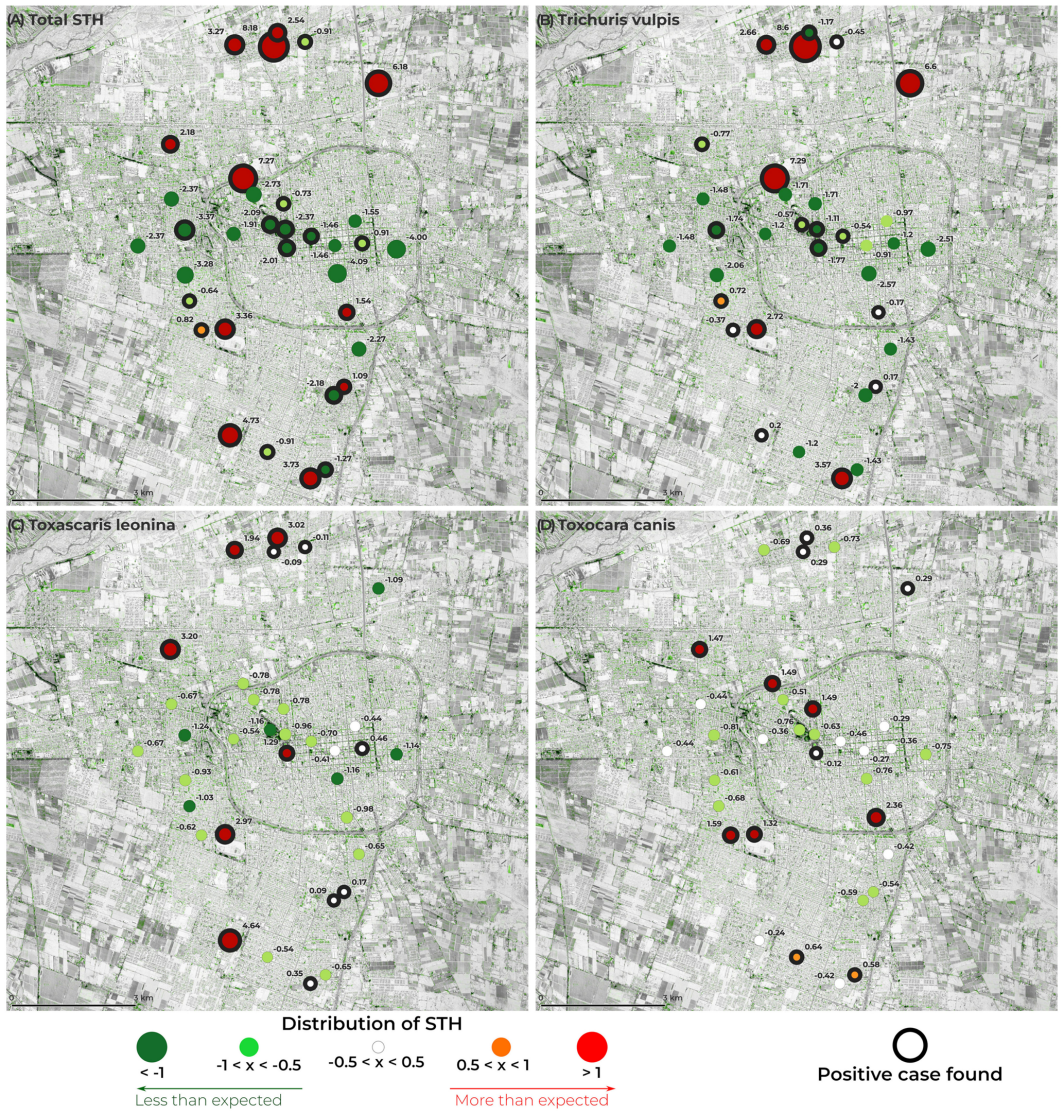
Distribution of canine soil-transmitted helminths (STH) in the study area of the city of San Juan (San Juan, Argentina). **(A)** Canine STH as a group. **(B)** *Trichuris vulpis*. **(C)** *Toxascaris leonina*. **(D)** *Toxocara* spp. Circles in red represent positive values where observed cases were higher than expected. Circles in green represent positive values where observed cases were less than the expected. In white, the difference between observed and expected values was not different. The circles with a black border represent those sites where positive samples were detected.

Additionally, the observation of the distribution of the three cSTH found in the different sampled areas, shows that they are heterogeneously and not homogeneously distributed (Figure 3A). This figure shows those areas where the presence of the parasites is either higher (red) or lower (green) than expected. The presence of *T. vulpis* (Figure 3B) was detected in 19 out of 32 (59.4%) of the parks and squares sampled; its presence was higher than expected in 5 of them. Although *T. leonina* was detected in 12 of the sites sampled (37.5%), its presence was higher than expected in 6 of these (Figure 3C). Like *T. leonina*, *Toxocara* spp. was found in 12 of the parks and squares samples and its presence was higher than expected in 6 of these sites (Figure 3D). The difference between observed and expected values, assuming a homogeneous distribution, for each sampled area, is presented in Table 2.

**Table 2:** The difference between observed and expected values, assuming a homogeneous distribution, of positive cases of canine soil-transmitted helminths (cSTHs) in canine environmental feces collected from different squares and parks from San Juan City (San Juan Province, Argentina).

Number	Difference from expected					Name	Total cSTH	*Trichuris vulpis*	*Toxascaris leonina*	*Toxocara* spp.
1	Plaza B° Ramos	2.54	−1.17	3.02	0.36
2	Plaza B° Chimbas	8.18	8.6	−0.09	0.29
3	Plaza Dep.Chimbas	−0.91	−0.45	−0.11	−0.73
4	Plaza B° Güemes	3.27	2.66	1.94	−0.69
5	Plaza B° Los Andes	6.18	6.6	−1.09	0.29
6	Plaza Barrio Aramburu	2.18	−0.77	3.2	1.47
7	Plaza Barrio del Carmen	7.27	7.29	−0.78	1.49
8	Plaza Ejército Argentino	−2.73	−1.71	−0.78	−0.51
9	Plaza Cementerio	−0.73	−1.71	−0.78	1.49
10	Parque de Mayo	−2.09	−0.57	−1.16	−0.76
11	Plaza España	−2.37	−1.11	−0.96	−0.63
12	Centro Cívico - Teatro	−2.01	−1.77	1.29	−0.12
13	Plaza Laprida	−1.46	−0.54	−0.7	−0.46
14	Plaza 25 de Mayo	−1.46	−0.91	−0.41	−0.27
15	Plaza Gertrudis Funes	−1.55	−0.97	−0.44	−0.29
16	Plaza Aberastain	−0.91	−1.2	0.46	−0.36
17	Zona Terminal	−4	−2.51	−1.14	−0.75
18	Plaza Hipólito Yrigoyen	−4.09	−2.57	−1.16	−0.76
19	Plaza Italia	−1.91	−1.2	−0.54	−0.36
20	Plaza Manuel Belgrano	−3.37	−1.74	−1.24	−0.81
21	Plaza Desamparados	−2.37	−1.48	−0.67	−0.44
22	Plaza Villa San Roque	−2.37	−1.48	−0.67	−0.44
23	Plaza Barrio Bancario	−3.28	−2.06	−0.93	−0.61
24	Pza. Barrio Foeva	−0.64	0.72	−1.03	−0.68
25	Pza. 2 Jardín Policial	0.82	−0.37	−0.62	1.59
26	Pza. Villa Sta. Anita	3.36	2.72	2.97	1.32
27	Plza Barrio La Estación	4.73	0.2	4.64	−0.24
28	Plaza Centenario	−0.91	−1.2	−0.54	0.64
29	Plaza B° San Ricardo	3.73	3.57	0.35	−0.42
30	Plaza Grillo	−1.27	−1.43	−0.65	0.58
31	Plaza Villa Fleuri	−2.18	−2	0.09	−0.59
32	Plaza Villa Lerga	1.09	0.17	0.17	−0.54
33	Plaza Echegaray	−2.27	−1.43	−0.65	−0.42
34	Plaza Almirante Brown	1.54	−0.17	−0.98	2.36

### 3.3. Weather data analysis

**Figure 4:**
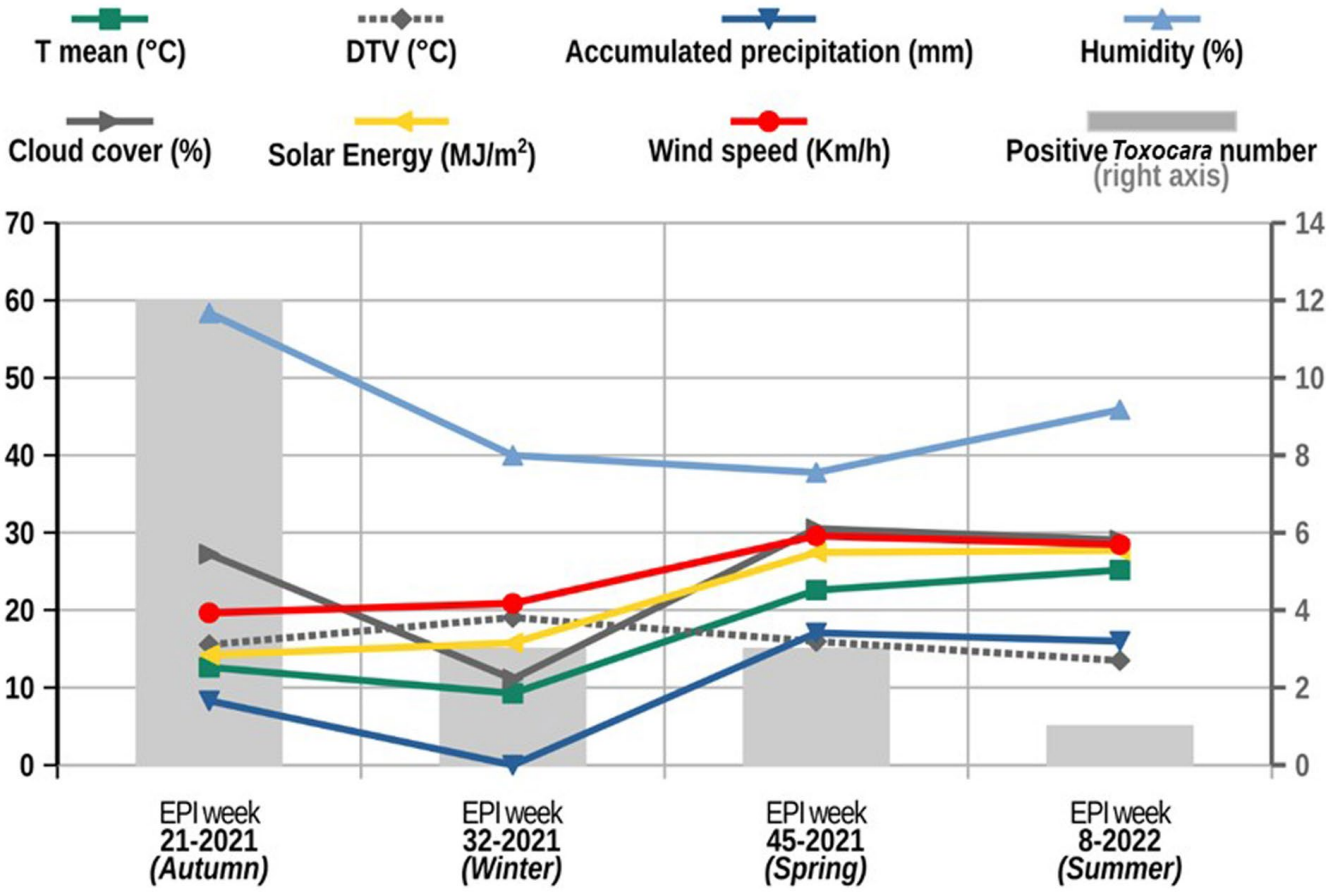
Description of the climatic variables retrieved from the weather station from San Juan Airport in San Juan (Argentina) and the number of *Toxocara* spp. cases found during the sample period (2021–2022). Climatic variables retrieved are shown on the left axis, including mean temperature (T mean in °C), Diurnal Temperature Variation (DTV), accumulated precipitation (mm), air humidity (%), cloud cover (%), solar energy (MJ/m^2^) and wind speed (km/h). The number of positive cases of *Toxocara* spp. found for each season (autumn, winter, spring and summer) is reflected on the right axis.

As previously stated, given that the detection of *Toxocara* spp. eggs was significantly more frequent during the autumn, the weather data was explored to identify any characteristics that might be driving this difference. The analysis of the different climatic variables (Figure 4) showed that for EW 21/2021, the air humidity was notably higher, while the wind speed and solar energy were somewhat lower. These weather features might be involved in the higher prevalence of *Toxocara* spp. eggs observed during the autumn given that high humidity, low wind speed and low solar radiation are a good combination of weather factors for the survival of *Toxocara* spp. eggs in the soil (28–32).

## 4. Discussion and conclusion

Through the sampling of fecal canine samples collected from 34 of the main urban parks and squares of the City of San Juan (San Juan, Argentina), the presence of different cSTH species was detected, including *Toxocara* spp., which is a zoonotic parasite that poses a risk to humans. The overall prevalence of cSTH found in this study was 8.9%, which is lower than the prevalence reported in other studies from urban areas in Argentina (33–36), and similar to the prevalence reported in Ushuaia (37) (Supplementary Table 1). Herein, the most prevalent cSTH was *T. vulpis*, while in other studies the most prevalent species found was *A. caninum* (33, 35, 38–40). The same pattern was observed in other countries such as Australia and Nigeria, where this hookworm species was the most prevalent (41, 42). In some of these studies, infection by protozoan species was higher than helminth infections (37); in this study, protozoan parasites were not detected, although the modified Ziehl-Neelsen technique (43), which is more sensitive, was not used.

In this study, *T. vulpis* was the most prevalent cSTH, this could be due to the longer survival time of eggs of this species in the soil; this might be increasing the chances for dogs that frequent the parks/squares to become reinfected (44). Moreover, *T. vulpis* has a prepatent period of 3 months, therefore antiparasitic treatment should be routinely repeated at monthly intervals to kill all the worms as they mature and prevent contamination of the environment (3). On the other hand, *Toxocara* spp. and *T. leonina* require only a few weeks to mature (1–2 months), and with a second dose of anthelmintic administered 2 or 3 weeks after the first one, the dogs would be free of all the worms. Considering that the samples analyzed herein are from the environment and that the status of each definitive host is unknown, we could assume that canine deworming is either not being performed or not given in periodic intervals.

Unfortunately, since fecal environmental samples were used, the association with characteristics of the dogs themselves (i.e., age, free-roaming or kept, underlying conditions, among others) (45) and with the conduct of care of the owners (i.e., antiparasitic treatment) (46) could not be considered. Moreover, the setting where this study was conducted was urban and the prevalence and variety of parasites found might be greater in rural areas where there is also exposure to other animals (4, 47, 48).

Additionally, differences in prevalence could also be due to the climatic and soil conditions of San Juan, given that it is an area with very low precipitation and other studies have shown that the average amount of rainfall was found to be strongly associated with the environmental contamination of parks with cSTH (41). Through the analysis of the association between the presence of *Toxocara* spp. and environmental characteristics, in this study, the regression analysis revealed that shadow significantly contributes to the increased prevalence of the parasites as measured by the TMI, as previously observed in other studies (28–32). The presence of trees and their shadows, along with other factors like irrigation and management of the park (not considered in this study), could create an ecological urban niche for the parasites to develop in the soil regardless of the general dry environment of San Juan. The significantly higher prevalence of *Toxocara* spp. observed in the autumn coincides with increased air humidity, lower wind speed and sun radiation, these environmental conditions could potentially facilitate transmission of *Toxocara* spp. eggs. This was confirmed under laboratory conditions in a previous study (32). In addition, other studies have shown that Argentina and Brazil have optimal humidity conditions for the development of *Toxocara* spp. eggs (49–51). Nevertheless, extreme temperatures (high or low) are also important as they can lead to desiccation of eggs and larval stages or arrested development of infective stages in the environment (52).

The cSTH species found herein were not homogeneously spread throughout the city, and there were areas that had conditions that were more appropriate for the transmission from one dog to another. In general, the cSTH detected in this study were found in the areas surrounding the Capital Department. Nonetheless, when analyzing per species, the prevalence of *Toxocara* spp. was greater than expected within the Capital Department, which is the most densely populated. These areas with the detection of a higher prevalence of *Toxocara* spp. than expected may be used to guide public health measures for screening of antibodies specific for *Toxocara* spp. in humans, especially children, given that Toxocariasis is a silent disease that could be acquired during infancy and have severe consequences (9). Further studies could be conducted to determine the possible risk factors associated with these areas (53). Unfortunately, evaluation of the egg’s viability and ability to become infective (54) was not performed, future studies must be conducted to evaluate these, given its implications on the risk to public health.

The regular administration of anthelmintic treatments and the promotion of responsible dog ownership, including picking up dog feces and hand hygiene are important measures which need to be adopted to minimize environmental contamination with *Toxocara* spp. and other STHs (41). Multidisciplinary research, formulated under “one health approaches” can deliver reinforced tools for exploring zoonotic parasites, including cSTHs (46).

Due to the low number of public squares and parks studied herein (*N* = 34), future studies with a higher number of squares and public parks should be conducted to improve the correlations analysis.

This is the first study in San Juan, Argentina to describe the presence of cSTH parasite species in public areas. The specific localization of squares and parks infected with cSTH eggs aim to provide information to design strategies to lower the cSTH infection burden in dogs and to provide information to direct serological screening of the human population, specifically for *Toxocara* spp. Given the zoonotic nature of these cSTHs we hope this information will help to reinforce activities of control programs, focusing on the “One Health” approach.

## Data availability statement

The original contributions presented in the study are included in the article/Supplementary material, further inquiries can be directed to the corresponding author.

## Author contributions

HA, VP, and MP: conceptualization. MP, HA, LS, PA, and VP: methodology. HA and MP: formal analysis. HA, PA, and SM: investigation. HA, LS, and MP: data curation. GG: statistical analysis. LS: spatial analysis. HA, GG, and LS: writing—original draft preparation. MP: writing—review and editing. VP and MP: supervision. All authors contributed to the article and approved the submitted version.

## Funding

This work was supported by Fundación Mundo Sano and Consejo Nacional de Investigaciones Científicas y Tecnológicas (CONICET).

## Conflict of interest

The authors declare that the research was conducted in the absence of any commercial or financial relationships that could be construed as a potential conflict of interest.

## Publisher’s note

All claims expressed in this article are solely those of the authors and do not necessarily represent those of their affiliated organizations, or those of the publisher, the editors and the reviewers. Any product that may be evaluated in this article, or claim that may be made by its manufacturer, is not guaranteed or endorsed by the publisher.

## Supplementary material

SUPPLEMENTARY FIGURE 1 Mean Tree Magnitude Index (TMI) of the study area of San Juan City (San 473 Juan, Argentina) during 2021. The squares and parks where the samples were collected are 474 marked in red.

## References

[1] Benedetti G Jokelainen P Ethelberg S. Search term "one health" remains of limited use to identify relevant scientific publications: Denmark as a case study. Front Public Health. (2022) 10:938460. doi: 10.3389/fpubh.2022.938460, PMID: 35968488 PMC9368311

[2] World Health Organization (2021). Tripartite and UNEP support OHHLEP’s definition of one health.” Available at: https://www.who.int/news/item/01-12-2021-tripartite-and-unep-support-ohhlep-s-definition-of-one-health (Accessed August 2, 2022).

[3] Bowman DD. Georgis’ Parasitology for Veterinarians. 10th ed Philadelphia: Saunders/Elsevier (2014).

[4] Zendejas-Heredia PA Crawley A Byrnes H Traub RJ Colella V. Zoonotic soil-transmitted helminths in free-roaming dogs, Kiribati. Emerg Infect Dis. (2021) 27:2163–5. doi: 10.3201/eid2708.204900, PMID: 34287132 PMC8314807

[5] Traversa D Frangipane di Regalbono A Di Cesare A La Torre F Drake J Pietrobelli M. Environmental contamination by canine geohelminths. Parasit Vectors. (2014) 1:67. doi: 10.1186/1756-3305-7-67, PMID: 24524656 PMC3929561

[6] Afarov A Mihalca AD Park GM Akramova F Ionică AM Abdinabiev O. A survey of helminths of dogs in rural and urban areas of Uzbekistan and the zoonotic risk to human population. Pathogens. (2022) 11:1085. doi: 10.3390/pathogens11101085, PMID: 36297142 PMC9610627

[7] Jenkins DJ. *Toxocara* canis in Australia. Adv Parasitol. (2020) 109:873–8. doi: 10.1016/bs.apar.2020.01.033, PMID: 32381231

[8] Jenkins EJ. *Toxocara* spp. in dogs and cats in Canada. Adv Parasitol. (2020) 109:641–53. doi: 10.1016/bs.apar.2020.01.026, PMID: 32381222

[9] Ma G Rostami A Wang T Hofmann A Hotez PJ Gasser RB. Global and regional seroprevalence estimates for human toxocariasis: a call for action. Adv Parasitol. (2020) 109:275–90. doi: 10.1016/bs.apar.2020.01.011 32381202

[10] Rostami A Riahi SM Holland CV Taghipour A Khalili-Fomeshi M Fakhri Y. Seroprevalence estimates for toxocariasis in people worldwide: a systematic review and meta-analysis. PLoS Negl Trop Dis. (2019) 13:e0007809. doi: 10.1371/journal.pntd.0007809, PMID: 31856156 PMC6922318

[11] Noordin R Yunus MH Tan Farrizam SN Arifin N. Serodiagnostic methods for diagnosing larval toxocariasis. Adv Parasitol. (2020) 109:131–52. doi: 10.1016/bs.apar.2020.01.003, PMID: 32381194

[12] Nijsse R Overgaauw P Ploeger H Mughini-Gras L. Sources of environmental contamination with *Toxocara* spp.: an omnipresent parasite. Adv Parasitol. (2020) 109:585–614. doi: 10.1016/bs.apar.2020.01.010, PMID: 32381219

[13] Deutz A Fuchs K Auer H Kerbl U Aspöck H Köfer J. *Toxocara*-infestations in Austria: a study on the risk of infection of farmers, slaughterhouse staff, hunters and veterinarians. Parasitol Res. (2005) 97:390–4. doi: 10.1007/s00436-005-1469-5, PMID: 16151740

[14] Etewa SE Abdel-Rahman SA Abd El-Aal NF Fathy GM El-Shafey MA Ewis AM. Geohelminths distribution as affected by soil properties, physicochemical factors and climate in Sharkyia governorate Egypt. J Parasit Dis. (2016) 40:496–504. doi: 10.1007/s12639-014-0532-5, PMID: 27413327 PMC4927514

[15] Rocha S Pinto RM Floriano AP Teixeira LH Bassili B Martinez A. Environmental analyses of the parasitic profile found in the sandy soil from the Santos municipality beaches, SP, Brazil. Rev Inst Med Trop Sao Paulo. (2011) 53:277–81. doi: 10.1590/s0036-46652011000500007, PMID: 22012454

[16] Kottek M Grieser J Beck C Rudolf B Rubel F. World map of the Köppen-Geiger climate classification updated. Meteorol Z. (2006) 15:259–63. doi: 10.1127/0941-2948/2006/0130

[17] Beck HE Zimmermann NE McVicar TR Vergopolan N Berg A Wood EF. Present and future Köppen-Geiger climate classification maps at 1-km resolution. Sci Data. (2018) 5:180214. doi: 10.1038/sdata.2018.214, Erratum in: Sci Data 2020 Aug 17;7(1):274 30375988 PMC6207062

[18] Dantas-Torres F Ketzis J Mihalca AD Baneth G Otranto D Tort GP. TroCCAP recommendations for the diagnosis, prevention and treatment of parasitic infections in dogs and cats in the tropics. Vet Parasitol. (2020) 283:109167. doi: 10.1016/j.vetpar.2020.109167, PMID: 32580071

[19] Smith GG. Diagnosing helminthiasis through Coprological examination. Can Vet J. (1981) 29:1021. doi: 10.4269/ajtmh.1980.29.1021

[20] Abou-El-Naga IF. Developmental stages and viability of *Toxocara canis* eggs outside the host. Biomedica. (2018) 38:189–97. doi: 10.7705/biomedica.v38i0.3684, PMID: 30184346

[21] Di Rienzo JA Casanoves F Balzarini MG Gonzalez LA Tablada ME Robledo CW (2019). InfoStat v. 2019. *Cent Transf InfoStat, FCA, Univ Nac Córdoba, Argentina*.

[22] Dean A. G. Sullivan KM Soe MM. (2013). OpenEpi: open source statistics for public health, Version 3.01. www.OpenEpi.com.

[23] Instituto Geográfico Nacional (IGN) (2017). Modelo Digital de Elevaciones Aerofotogramétrico de Cuyo. Available at: https://www.ign.gob.ar/NuestasActividades/InformacionGeoespacial/Principal (Accessed August 2, 2022).

[24] Gorelick N Hancher M Dixon M Ilyushchenko S Thau D Moore R. Remote sensing of environment Google earth engine: planetary-scale geospatial analysis for everyone. Remote Sens Environ. (2017) 202:18–27. doi: 10.1016/j.rse.2017.06.031

[25] Visual Crossing Corporation. Visual crossing weather (2021-2022). Available at: https://www.visualcrossing.com/resources/documentation/weather-data/how-to-i-properly-cite-or-attribute-the-weather-data-in-my-project-to-visual-crossing-weather/ (Accessed September 5, 2022).

[26] Brunaská M Dubinský P Reiterová K. *Toxocara canis*: ultrastructural aspects of larval moulting in the maturing eggs. Int J Parasitol. (1995) 25:683–90. doi: 10.1016/0020-7519(94)00183-o, PMID: 7657453

[27] el Naga IF. *Toxocara canis*: determination of the origin of antigenic materials released from infective larvae. J Egypt Soc Parasitol. (2000) 30:669–78. 11198365

[28] Erofeeva VV Vasenev V. Influence of environmental factors on the development and survival of *Toxocara* Sp. eggs in various soil substrates In: Vasenev V Dovletyarova E Cheng Z Valentini R Calfapietra C, editors. Green Technologies and Infrastructure to Enhance Urban Ecosystem Services. SSC 2018. Cham: Springer Geography, Springer (2020)

[29] Azam D Ukpai OM Said A Abd-Allah GA Morgan ER. Temperature and the development and survival of infective *Toxocara canis* larvae. Parasitol Res. (2012) 110:649–56. doi: 10.1007/s00436-011-2536-8, PMID: 21779864

[30] Gao X Wang H Li J Qin H Xiao J. Influence of land use and meteorological factors on the spatial distribution of Toxocara canis and Toxocara cati eggs in soil in urban areas. Vet Parasitol. (2017) 233:80–5. doi: 10.1016/j.vetpar.2016.12.004, PMID: 28043392

[31] Vargas Nava AI Castro Del Campo N EnrÍquez Verdugo I Portillo Loera JJ Barraza Tizoc CL Gaxiola Camacho SM. Prevalence and viability of *Toxocara* spp. eggs in soil of public parks in northwestern Mexico. Iran J Parasitol. (2020) 15:196–203. doi: 10.18502/ijpa.v15i2.3301 32595709 PMC7311814

[32] Gamboa MI. Effects of temperature and humidity on the development of eggs of *Toxocara canis* under laboratory conditions. J Helminthol. (2005) 79:327–31. doi: 10.1079/joh2005287, PMID: 16336716

[33] Gamboa M Corbalán V Paladini A Butti M Osen B Carabajal R. Zoonosis parasitarias en caninos de un área vulnerable. Rev Enfermedades Infecc Emergentes. (2020) 15:39–44.

[34] Soriano SV Pierangeli NB Roccia I Bergagna HF Lazzarini LE Celescinco A. A wide diversity of zoonotic intestinal parasites infects urban and rural dogs in Neuquén, Patagonia, Argentina. Vet Parasitol. (2010) 167:81–5. doi: 10.1016/j.vetpar.2009.09.048, PMID: 19864068

[35] Taranto NJ Passamonte L Marinconz R De Marzi MC Cajal SP Malchiodi EL. Parasitosis zoonoticas transmitidas por perros en el Chaco Salteño. Medicina. (2000) 60:217–20. PMID: 10962811

[36] Sánchez P Raso S Torrecillas C Mellado I Ñancufil A Oyarzo CM. Contaminación biológica con heces caninas y parásitos intestinales en espacios públicos urbanos en dos ciudades de la Provincia del Chubut: Patagonia Argentina. Parasitol Latinoam. (2003) 58:131–5. doi: 10.4067/S0717-77122003000300008

[37] Cociancic P Deferrari G Zonta ML Navone GT. Intestinal parasites in canine feces contaminating urban and recreational areas in Ushuaia (Argentina). Vet Parasitol Reg Stud Rep. (2020) 21:100424. doi: 10.1016/j.vprsr.2020.100424, PMID: 32862914

[38] Andresiuk V Rodríguez F Denegri G Sardella N Hollmann P. Relevamiento de parásitos zoonóticos en materia fecal canina y su importancia para la salud de los niños. Arch Argent Pediatr. (2004) 102:325–9.

[39] Rodríguez F Denegri G Sardella N Hollmann P. Relevamiento coproparasitológico de caninos ingresados al Centro Municipal de Zoonosis de Mar del Plata, Argentina. Rev Vet. (2005) 16:9.

[40] La Sala LF Leiboff A Burgos JM Costamagna SR. Spatial distribution of canine zoonotic enteroparasites in Bahía Blanca, Argentina. Rev Argent Microbiol. (2015) 47:17–24. doi: 10.1016/j.ram.2014.12.006, PMID: 25705047

[41] Massetti L Wiethoelter A McDonagh P Rae L Marwedel L Beugnet F. Faecal prevalence, distribution and risk factors associated with canine soil-transmitted helminths contaminating urban parks across Australia. Int J Parasitol. (2022) 52:637–46. doi: 10.1016/j.ijpara.2022.08.001, PMID: 36007621

[42] Jajere SM Lawal JR Shittu A Waziri I Goni DM Fasina FO. Epidemiological study of gastrointestinal helminths among dogs from northeastern Nigeria: a potential public health concern. Parasitol Res. (2022) 121:2179–86. doi: 10.1007/s00436-022-07538-z, PMID: 35543746

[43] Current WL. Techniques and laboratory maintenance of cryptosporidium In: . Cryptosporidiosis of Man and Animals eds. J. P. Dubey, C. A. Speer and R. Fayer (Boca Raton: CRC Press) (1990)

[44] Traversa D. Are we paying too much attention to cardio-pulmonary nematodes and neglecting old-fashioned worms like *Trichuris vulpis*? Parasit Vectors. (2011) 4:32. doi: 10.1186/1756-3305-4-32, PMID: 21385441 PMC3063211

[45] Byaruhanga C Knobel D. Sex as a risk factor for occurrence and severity of infectious and parasitic diseases in dogs: protocol for a systematic review. PLoS One. (2022) 17:e0275578. doi: 10.1371/journal.pone.0275578, PMID: 36282817 PMC9595549

[46] Colella V Traub RJ Gasser RB. Translational research of zoonotic parasites: toward improved tools for diagnosis, treatment and control. Pathogens. (2021) 10:1416. doi: 10.3390/pathogens10111416, PMID: 34832572 PMC8621207

[47] Enriquez GF Macchiaverna NP Argibay HD López Arias L Farber M Gürtler RE. Polyparasitism and zoonotic parasites in dogs from a rural area of the argentine Chaco. Vet Parasitol Reg Stud Rep. (2019) 16:100287. doi: 10.1016/j.vprsr.2019.100287, PMID: 31027600

[48] Zhang X Jian Y Ma Y Li Z Fu Y Cairang Z. Prevalence of intestinal parasites in dog Faecal samples from public environments in Qinghai Province, China. Pathogens. (2022) 11:1–8. doi: 10.3390/pathogens11111240 PMC9696243 36364990

[49] Bojanich MV Alonso JM Caraballo NA Itatí Schöller M López Mde L García LM. Assessment of the presence of *Toxocara* eggs in soils of an arid area in Central-Western Argentina. Rev Inst Med Trop Sao Paulo. (2015) 57:73–6. doi: 10.1590/S0036-46652015000100010, PMID: 25651329 PMC4325526

[50] Dantas-Torres F Otranto D. Dogs, cats, parasites, and humans in Brazil: opening the black box. Parasit Vectors. (2014) 7:22. doi: 10.1186/1756-3305-7-22, Erratum in: Parasit Vectors. 2016;9(1):298 24423244 PMC3914713

[51] Santarém VA Franco Eda C Kozuki FT Fini D Prestes-Carneiro LE. Environmental contamination by *Toxocara* spp. eggs in a rural settlement in Brazil. Rev Inst Med Trop Sao Paulo. (2008) 50:279–81. doi: 10.1590/s0036-46652008000500006, PMID: 18949345

[52] Dybing NA Fleming PA Adams PJ. Environmental conditions predict helminth prevalence in red foxes in Western Australia. Int J Parasitol Parasites Wildl. (2013) 2:165–72. doi: 10.1016/j.ijppaw.2013.04.004, PMID: 24533331 PMC3862530

[53] Na-Ek P Narkkul U Phasuk N Punsawad C. Seroprevalence of anti-*Toxocara canis* antibodies and associated risk factors among dog owners in the rural community of Nakhon Si Thammarat province, southern Thailand. Trop Med Health. (2022) 50:32. doi: 10.1186/s41182-022-00425-4, PMID: 35581656 PMC9112435

[54] Otero D Alho AM Nijsse R Roelfsema J Overgaauw P Madeira de Carvalho L. Environmental contamination with *Toxocara* spp. eggs in public parks and playground sandpits of greater Lisbon, Portugal. J Infect Public Health. (2018) 11:94–8. doi: 10.1016/j.jiph.2017.05.002, PMID: 28545900

