# Supplementary figures and images for: Environmental Toxocara spp. presence in crowded squares and public parks from San Juan Province, Argentina: A call for a “One Health” approach
Source: Front Med (Lausanne). 2023 Feb 17;10:1102396. doi: 10.3389/fmed.2023.1102396 (PMC9982091; doi:10.3389/fmed.2023.1102396)

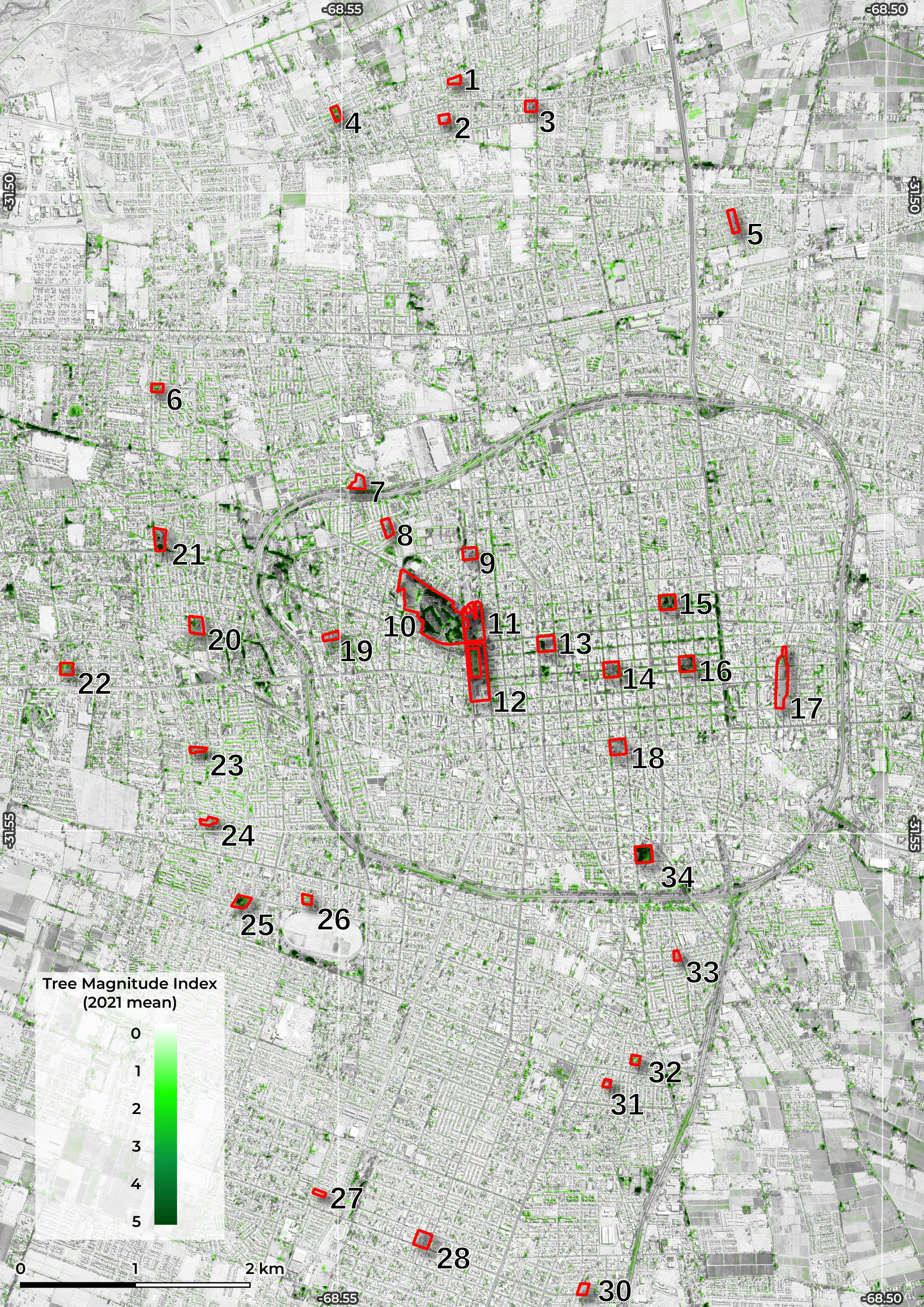

Supplement: SUPPLEMENTARY FIGURE 1 — Mean Tree Magnitude Index (TMI) of the study area of San Juan City (San 473 Juan, Argentina) during 2021. The squares and parks where the samples were collected are 474 marked in red. [file Image_1.JPEG]
