# Supplementary material for: Environmental Toxocara spp. presence in crowded squares and public parks from San Juan Province, Argentina: A call for a “One Health” approach
Source: Front Med (Lausanne). 2023 Feb 17;10:1102396. doi: 10.3389/fmed.2023.1102396 (PMC9982091; doi:10.3389/fmed.2023.1102396)
Supplement: Supplementary file 2 [file Table_1.docx]

| **Province** | **Area** | **Method*** | ***Trichuris vulpis***  **Prevalence (%)** | ***Toxocara spp.***  **Prevalence (%)** | **Toxascaris leonina**  **Prevalence (%)** | **Cite** |
| --- | --- | --- | --- | --- | --- | --- |
| Buenos Aires | Peri-Urban | Sheather & Telemann | 17.8 | 23.2 | 0 | (33) |
|  | Urban | Willis & Telemann | 48.9 | 1 | 0 | (38) |
|  | Urban | Willis & Telemann | 52.19 | 6.83 | 0 | (39) |
|  | Urban | Telemann | 18.1 | 2.3 | 0 | (40) |
| Chaco | Rural | Willis | 3.5 | 14.1 | 0 | (47) |
| Neuquén | Rural | Sheather &Telemann | 3.26 | 41.15 | 2.11 | (34) |
|  | Urban |  | 46.75 | 48.10 | 0 |  |
| Salta | Rural | Willis & Telemann | 7.5 | 17.2 | 0 | (35) |
| Chubut | Urban | Willis & Telemann | 0 | 0.19 | 0 | (36) |
| Tierra del Fuego | Urban | Telemann & FLOTAC® | 0 | 5.0 | 1.3 | (37) |

**Supplementary Table 1**: Studies of dog feces parasite performed in Argentina.

* Sheather method (saturated sugar solution, 1.25 specific gravity), Willis method (saturated NaCl solution, 1.20 specific gravity), Telemann method (sedimentation technique), FLOTAC® (quantitative method, saturated NaCl solution, 1.20 specific gravity).
